# Establishing Digital Biomarkers for Occupational Health Assessment in Commercial Salmon Fishermen: Protocol for a Mixed-Methods Study

**DOI:** 10.2196/10215

**Published:** 2018-12-10

**Authors:** Rachel Elizabeth Wilbur, Jacob Spencer Griffin, Mark Sorensen, Robert Daniel Furberg

**Affiliations:** 1 Department of Anthropology University of North Carolina Chapel Hill, NC United States; 2 RTI International RTP, NC United States

**Keywords:** digital health, digital biomarkers, occupational health, health and safety, fishermen, physiological health, stress

## Abstract

**Background:**

Commercial salmon fishing in Alaska is one of the most dangerous occupations in the United States. Between 1992 and 2008, the average annual industry mortality rate was 128 deaths per 100,000 workers, and despite an increase in industry regulations, there has not been a significant decrease in mortality rate since 2000. Unpredictable fishing openings and fierce competition for limited resources result in periods of intense sleep deprivation and physical strain during the short commercial salmon season in Alaska.

**Objective:**

We hypothesize that the combined effect of sleep deprivation, intense physical workload, and significant short-term chronic stress may be deleterious to health in both the short- and long-term among commercial salmon drift gillnet fishermen in Alaska. The objective of this protocol is to determine the feasibility of the study design to test this hypothesis.

**Methods:**

The study design uses mixed methods and includes biometric monitoring consisting of heart rate variability, respiration, and movement data collected via a personal, wearable biometric device. Additional methods include observational data on activity, including duration and quality of sleep, weather, catch, and financial gain, as well as the collection of salivary cortisol. As such, the study will provide a holistic assessment of individual stress on multiple simultaneous timescales: immediately and continuously through the personal wearable biometric device, on the minute-hour level through the multiple daily collections of salivary cortisol, and by the hour-day through the use of participant and environment observational data.

**Results:**

Data collection was initiated in July 2017 and will extend through August 2019. Initial data collection has indicated that the methods outlined in this protocol are feasible and allow for effective collection of qualitative and quantitative data related to the psychological and physiological impact of Alaska commercial salmon fishing.

**Conclusions:**

We anticipate that the use of a biometric device will be crucial in establishing measures of stress and physical activity within a population and environment uniquely challenged by physical isolation, strong weather patterns, and the potential for significant financial gain by fishermen. The potential exists for individuals engaged long-term in the fishing industry, through repeated and extended exposure to periods of intense sleep deprivation and chronic stress, to be at increased risk of cardiovascular disease.

**International Registered Report Identifier (IRRID):**

DERR1-10.2196/10215

## Introduction

### Background

Commercial salmon fishing in Alaska is one of the most dangerous occupations in the United States. Between 1992 and 2008, the average annual industry mortality rate was 128 deaths per 100,000 workers [1], and despite an increase in industry regulations, there has not been a significant decrease in mortality rate since 2000 [2]. Comparatively, the next most dangerous civilian occupations, as of 2016, are aircraft pilot, roofer, truck driver, and farmer, with rates of 55.5, 48.6, 24.7, and 23.1 fatalities per 100,000 workers, respectively [3]. Given the seasonal and often isolated nature of the work, reliable statistics on injuries are unavailable. The majority of recorded injuries and mortality result from vessel disasters, on-deck injuries, particularly those involving machinery, and falls overboard [2,4]. The industry is calling for injury prevention efforts such as increased use of personal flotation devices, but efforts to date have proven minimally successful.

Studies have demonstrated a significant, positive relationship between sleep deprivation and response time and vigilant attention [5]. Sleep deprivation has also been shown to decrease resilience to stress and have a detrimental impact on complex reasoning and decision making [6]. Unpredictable fishing openings and fierce competition for limited resources result in periods of intense sleep deprivation and physical strain during the short commercial salmon seasons in Alaska. For example, in the 24 hours from July 15-16, 2018, there were a total of 19 hours open for fishing in two 9.5-hour periods and only 5 hours during which fishing was not allowed (referred to in the industry as a closure), also in two 3-hour periods. Given the average 5-week season, most fishermen find it imperative to maximize fishing time. Sleep, therefore, most often occurs during short closures, which are typically also used to deliver fish, clean, and mend gear, resulting in sleep patterns which resemble naps rather than true sleep. The National Institutes of Health recommends that healthy adults receive between 7 and 8 hours of uninterrupted sleep per night [7]. For the purposes of this study, and in keeping with the American Academy of Sleep Medicine, sleep deprivation is defined as a pattern of sleep that results in excessive daytime sleepiness, mood changes, performance issues, and poor health outcomes [8].

The work of commercial gillnet salmon fishing is often highly physically demanding, requiring regular lifting and pulling of heavy items and repetitive movements associated with picking fish from nets, which is performed at high speeds and often for hours at a time. While the industry is currently focused on reducing acute morbidity and mortality, chronic stress, sleep deprivation, and the maintenance of high levels of physical exertion may also contribute to morbidity later in life through increased cardiometabolic risk [9]. We hypothesize that the combined effect of sleep deprivation, intense physical workload, and significant short-term chronic stress may be deleterious to health in both the short- and long-term among commercial salmon drift gillnet fisherman in Alaska.

In order to identify the physical and physiological impacts of commercial fishing on individuals, we intend to complete a mixed-methods study designed to elucidate the ways in which sleep deprivation, poor sleep quality, and chronic stress impact the short- and long-term health of industry participants. Advancements in biometrics of activity, movement, and physiology in combination with biomarkers of acute and chronic psychosocial stress can provide insight into how the lifestyle and occupational factors associated with commercial fishing may negatively impact both the physical and psychological health of fishermen, while observational data can provide context into the daily activities and environmental pressures inherent to the industry.

Biometric devices provide an objective method for quantifying physical activity. Measuring physical activity and sleep deprivation has proven historically challenging and traditionally has been performed using self-report via physical activity questionnaires. While cheap and relatively easy to administer [10,11], physical activity questionnaires are incapable of objectively quantifying physical activity and, due to self-report, are inherently unreliable. One study in 2011 found a 40% difference between participants who met recommended daily activity levels when daily activity levels were determined through self-reported questionnaire versus direct researcher observation [12]. Objective methods are necessary to quantify physical activity in order to assess its impacts on health.

Recent advancements in personal activity tracking devices promise a solution to the issue of subjectivity. The use of accelerometers and heart rate monitors to record energy expenditure and other measures of physical activity is not new, but improved technology, including media sharing, has rendered the products more reliable, reducing cost and increasing their popularity among consumers [13]. Modern personal tracking devices have been found to have a high level of accuracy and interdevice reliability [11,13-16] and provide an objective measure of physical activity [11,13-15]. While research-grade devices have been more thoroughly validated in laboratory settings, the recent advancements in commercially available biometric devices provides a novel and cost-effective approach for field research due to their ease of application. Further validation studies are needed to assess the reliability and accuracy of these devices in research settings. Studies have shown that only a short period of use is required in order to get an accurate assessment of an individual’s activity due to natural daily fluctuations in exercise. A recent study found that wearing an accelerometer for only 3 days was sufficient to accurately reflect activity [17]. This reduces the potential inconvenience caused by wearing such a device for an extended period of time. Although personal wearable tracking devices have demonstrated advantages for collecting measures of physical activity in individuals, little research has been done on their utility within an environment as complex and dangerous as commercial fishing. We anticipate that the use of a biometric device will be crucial in establishing measures of stress and physical activity within a population and environment uniquely challenged by physical isolation, strong weather patterns, and the potential for significant financial gain by fisherman.

While the collection of biometric data would enable us to monitor the body’s physical response to external stressors, the additional collection of the stress hormone cortisol allows for the measurement of the body’s chemical responses to the same stimuli. Cortisol, a hormone of the hypothalamic- pituitary-adrenal (HPA) axis, is produced by the adrenal glands in response to stress. Cortisol follows a circadian rhythm during which levels peak 30 to 45 minutes after waking and then decline throughout the remainder of the day [18-21]. In a normal state, the HPA axis responds to psychosocial stress through increasing cortisol production [22-27]. Cortisol plays an important role in maintaining cellular homeostasis through diverting metabolic operations toward systems at risk of dysregulation [22,24,25,27]. In healthy individuals, cortisol suppresses immune response and prevents increasing levels of proinflammatory cytokines [27-30]. Chronic stress causes a dysregulation of the HPA axis resulting in blunted cortisol reactivity [25,29,30]. A significant, negative correlation between the number or intensity of psychosocial stressors and cortisol levels has been shown in individuals with posttraumatic stress disorder, work-related stress, and traumatic events early in life [25,29]. The exact mechanism through which this dysregulation occurs is not well understood as it may be regulated through the reduction of cortisol production or the insensitivity of glucocorticoid receptors on target tissues [25,27]. Cortisol is a glucocorticoid hormone that activates the pathways responsible for preventing the release of proinflammatory cytokines in response to environmental, psychosocial, and physiological stress and is the most commonly used biomarker to assess levels of acute and chronic physiological stress [20,21,31-33]. Cortisol is a useful biomarker because it can be measured through saliva, allowing for noninvasive measurement of physiological stress response throughout the day.

While biomarker data is integral to understanding the human response to stress, observational data collected through participant observation provides an environmental context that is otherwise missing with the collection of biomarkers alone. Participant observation is a method of research used extensively within social science disciplines [34]. It involves the observation and recording of participant activities within their daily environment, instead of an artificial research environment, and has been found to be particularly useful in community health research [35-37]. Participant observation is often preferred to survey data collection due to improvements in reliability and recall and has the added benefit of allowing the observer to record external environmental conditions which may impact the participant’s behavior [35]. Mixed-methods studies, which include the use of both quantitative and qualitative data, in this case biometric, observational, and biomarker, can provide a more holistic perspective of health.

### Short-Term Health Impacts

Short-term health risks associated with sleep deprivation align most closely with industry safety concerns. Sleep deprivation, which results from an inadequate quantity or quality of sleep, has been shown to result in an increased risk of acute injury resulting from delayed response times. In addition, it has been shown to retard decision-making capabilities [38]. These incidences of injury and mortality result in the statistics which make the industry one of the most dangerous in the country.

### Long-Term Health Impacts

The short-term poor health outcomes resulting from commercial fishing are likely to cause the greatest concern to industry leaders and occupational health providers due to the stark image the statistics present. The long-term health impacts of combined sleep deprivation and intense physical and psychological stress, however, may result in a myriad of chronic diseases that have the potential to significantly negatively impact later life fitness and quality of life and potentially contribute to premature mortality. Acute sleep deprivation has been shown to impact control of cardiovascular regulation while altering inflammatory regulatory response and endothelial function [39], and short sleep duration has been found to be associated with numerous poor health outcomes including cardiovascular disease [40]. While the impact of short sleep duration seems to increase with age, even healthy early adults demonstrated significant effects [41,42]. Studies of the long-term impacts of sleep deprivation are inconclusive due in large part to experimental differences in environment, timing, and study population. Elevated blood pressure and hypertension have long been associated with increased risk of morbidity and disability as well as mortality from causes such as cardiovascular disease and stroke [41,43]. The relationship between sleep processes and the cardiovascular system are likely bidirectional, as poor cardiovascular health can result in disturbed sleep from causes like obstructive sleep apnea [44].

As with sleep deprivation, experiencing chronic stress has long-term health implications including memory impairment, increased abdominal obesity, and elevated risk of cardiovascular disease. Chronically elevated cortisol levels have been significantly associated with memory impairment in healthy adults [45] and have been demonstrated to interfere with attention and working memory in several experimental studies [45-48]. Besides detrimental effects to memory, variation in cortisol levels is associated with increased risk for abdominal obesity and metabolic dysregulation through perturbations of the HPA axis [19]. Persistent dysregulation of the HPA axis can result in a blunted cortisol response to stimuli, which has been linked to imbalances between proinflammatory and anti-inflammatory activity in the body [49]. This results in heightened levels of inflammatory cytokines, such as c-reactive protein and interleukin-6, that influence the etiology of cardiovascular disease and are predictors of cardiac episodes [49]. A similar relationship to inflammation has been found with interrupted sleep [50-52]. Within the general adult population, coronary heart disease and stroke are two of the leading contributors to morbidity and mortality in the United States [53]. The most recent report from the American Heart Association estimates that one American dies every 40 seconds from complications related to cardiovascular disease [53]. In 2013, 1 in every 9 deaths was attributed to myocardial infarction [53]. The potential therefore exists for individuals engaged long-term in the fishing industry, through repeat and extended exposure to periods of intense sleep deprivation and chronic stress, to be at increased risk of cardiovascular disease.

### Study Objective

The objective of this study is to determine the feasibility of using a wearable biometric device in combination with observational data and biomarkers of acute stress to assess the potential short- and long-term negative health impacts associated with Alaska commercial salmon gillnet fishing. The use of a personal wearable biometric device in tandem with salivary cortisol will allow us to collect continuous measures of physical exertion and stress in an infamously challenging environment and under austere conditions on 2 distinct time scales: biometric measures capture changes in the millisecond-to-second time scale while salivary cortisol records changes by the minute to hour. It also affords an accuracy that would not be possible with self-report surveys. The completion of this protocol will determine the feasibility of a mixed-methods study approach using a wearable biometric device, cortisol collection, and participant observation to answer crucial questions regarding safety and health impacts in a notoriously challenging population and occupation.

## Methods

### Study Design

This pilot study is intended to determine the feasibility of using a wearable biometric garment and salivary biomarkers (cortisol) coupled with qualitative data collection as measures of physiological and psychological occupational stress in commercial gillnet salmon fishermen in Alaska. A significant strength of this study design is that it will allow for a holistic assessment of individual stress on multiple simultaneous time scales: immediately and continuously through the personal wearable biometric device, on the minute-hour level through the multiple daily collections of salivary cortisol, and by the hour-day level through the use of observational data collection. We will use nonprobability convenience-based sampling to recruit 10 participants from the Bristol Bay, Alaska, commercial fishing fleet.

### Recruitment

Fliers advertising the study will be posted in one of the main boat yards where Bristol Bay driftnet and setnet gillnet fishing vessels are stored and maintained, including the main office, bathrooms, and fishermen’s lounge. Interested participants will be encouraged to contact the primary investigator via phone. Additional study information will be shared at this time, and if the potential participant retains interest in the study, an in-person meeting at a neutral location will be arranged. During the meeting, participant and study staff will discuss the study’s goals, potential risks to participants, expectations for wearing the biometric measurement device, compensation structure, study schedule, and our inability to provide absolute confidentiality. We will stress that this is not a medical study. At this meeting, we will also demonstrate the use of the biometric measurement device procedures to the participant. The recruitment goal for this leg of the study will be capped at 10 participants in order to confirm feasibility of the protocol.

### Selection Criteria

Recruitment will be limited to captains and crew members with a Bristol Bay, Alaska, commercial salmon drift gillnet license or crew license. At this phase of the feasibility study, recruitment will be limited to males between the ages of 18 and 50 years in order to maximize comparability of results from participants.

### Ethics and Confidentiality

This study has been reviewed and approved by the University of North Carolina (UNC) Institutional Review Board.

### Data Collection

#### Biometric Monitoring

Technological advances in wearable sensory devices have made it possible to gather extensive biometric data from subjects with minimal discomfort or researcher interference. Three key biometric measures are of particular interest when studying physiological stress response in ambulatory settings: accelerometry, heart rate variability (HRV), and respiratory analysis. HRV is calculated using variation in time between 2 heartbeats and provides a measure of the heart’s ability to respond to regulatory impulses. It can therefore act as an indicator of changes in stress [54].

Biometric data will be collected using Hexoskin (Carre Technologies Inc) technology in order to measure and record physical activity, heart rate, respirations, and sleep quality. In laboratory settings, Hexoskin technology has been found to have low variability and good agreement and consistency [55], although results of field testing have been mixed [56]. We believe that the Hexoskin design, as a form-fitting and moisture-wicking shirt, will provide less interference in the required daily activities of active fishermen. The devices have previously been found to be effective at collecting biometric measures in other highly active participants, including hikers and elite cyclists [56,57]. Similar products have been used to study occupational stress in firefighters and policemen, but to our knowledge, there have been no studies which made use of similar technologies to study commercial fishermen. The combined measures of HRV, respiration, and movement present a proxy for physical and psychological stress, as the heart rate increases in response to stressors through the fight-or-flight reflex. We use Hexoskin, an electrocardiograph (ECG) device, in order to capture HRV through variation in the R–R interval. HRV has been identified as an important measure of physical stress and activity and is not captured by photoplethysmograph (PPG) devices. Due to the challenging field environment in which we propose this research taking place, we determined that use of the Hexoskin would be the most applicable due to its comfort of wear and ease of data extraction while in an off-the-grid field environment [56].

After training performed by the primary investigator, the participant will wear the biometric device for a total of 48 hours prior to the initiation of the fishing season, in order to determine baseline measures. Every 12 hours, the device will be removed in order for the data to be uploaded to a database and the device’s battery to be recharged. Following the collection of baseline measures, the participant will wear the device for a total of 48 hours during the peak of the commercial sockeye salmon fishing season. As during the baseline measurement period, the device will be removed every 12 hours so the data can be uploaded and the batteries can be charged.

#### Cortisol

Salivary cortisol will be collected 5 times per day over the course of 2 days prior to the initiation of the fishing season in order to create a diurnal baseline before the introduction of extreme stressors associated with the fishing season. Before the study begins, the participants will be trained on how to collect samples according to the procedures [58]. Participants will be asked to collect their samples upon waking, 30 minutes postwaking, before lunch, in the evening, and prior to sleep using the passive drool technique. The collection vials will all be labeled before collection begins to avoid any commingling or mislabeling of specimens. All samples will be frozen and packed in ice before they are brought back to the UNC Human Biology laboratory for analysis. Using the same procedures, the participant will collect salivary cortisol for 3 days during the peak of the commercial sockeye salmon fishing season. Due to the erratic and inconsistent sleep patterns during the fishing season, the participant will not be able to provide salivary samples at the same 5 times each day. Instead, the participant will collect their saliva samples at approximately every 5 hours, with flexibility in specimen collection time when needed to allow for sleep.

#### Observational Data

During periods of biometric data collection, the primary investigator will observe the participants and make note of key events in their day and the time at which they occurred, including but not limited to waking, sleeping, eating, opening times for fishing, fishing initiation, time spent setting the net, net collection, fish picking, and travel. The investigator will also note factors such as weather and the quantity and weight of catch. Quantity and weight of catch will be verified using industry catch tickets, provided to each captain as a receipt upon the delivery of each catch. Observational data will be recorded using time-demarcated spreadsheets and will ultimately be used to calculate total hours of sleep per day. Given the variability of commercial fishing openings, sleep patterns during the season are often sporadic, with fisherman typically sleeping in shifts and for short periods of time throughout the day (investigator observation). Data recorded by the Hexoskin device during periods identified as sleep will be used to determine sleep quality.

### Data Analysis

The 3 data sources will be assimilated and synchronized in order to produce a filtered and preprocessed dataset for each participant suitable for statistical analysis. Fishing period data will be compared to baseline data as the control for analytical purposes.

#### Accelerometry

Accelerometry will be quantified using the physical activity index developed by Bai and colleagues [59]. This new metric uses the variance of oscillations along all 3 axes to calculate summary measures using predetermined epochs. ActivityIndex software (R Foundation for Statistical Computing) will be used to calculate the physical activity index from the raw accelerometry data collected from the Hexoskin.

#### Heart Rate Signal Processing and Variability

HRV refers to the variation in time between consecutive heartbeats over a specific amount of time. Changes in the time interval reflects periods of stress due to activation of the autonomic nervous system. While there remains ongoing research into the contributions of the sympathetic and parasympathetic nervous systems on HRV, its utility as a tool to detect changes in cardiac autonomic regulation is well established [60]. HRV is a popular measure in part due to noninvasive collection procedures and the plethora of computer-based analytical tools available [61]. HRV collection will be recorded for 3 phases—baseline, event, and postevent—according to recommended standards. This will allow the researchers to measure both the reactivity and recovery of HRV following both stressful and strenuous events that occur during the fishing season. Reactivity refers to the changes between the tonic baseline HRV and phasic stimulus-response HRV. Assessing the phasic HRV within the context of the activity is needed in order to determine whether the response is adaptive or not [54]. In this study, HRV will be filtered to reduce variations in signal by smoothing the HRV time series, and the ECG will be preprocessed in order to address noise interference by using a low-pass and high-pass filter and differentiator [62]. Participant average parameters will be calculated based on baseline data, and periods of stress will be identified by calculating mean and standard deviations within preset periods of time. HRV will be analyzed using the open source RHRV package (R Foundation for Statistical Computing).

#### Statistical Analysis of Heart Rate Variability

Before analyzing HRV, we will use ECG sensors to examine the electrical activity of study participants as the heart depolarizes. As the biometric monitoring device does not include all 10 electrodes required for a full ECG, this will constitute a semicomplete ECG. R-Project Statistical Software (R Foundation for Statistical Computing) will then be used for analysis.

#### Respiratory Analysis

Baseline average respiratory rate will be calculated from respirations measured during the control period prior to the initiation of fishing. Respiratory data collected during the peak of the season will be compared using VivoSense software version 3.0 (Vivonoetics) to determine periods when the participant’s respiratory rate was significantly different from baseline.

### Statistical Analysis of Observational Data

Observational data will be analyzed using Dedoose software version 8.0 (SocioCultural Research Consultants) to identify key periods of change. These periods are graphically represented according to the period of time during which they occurred and will be graphically overlaid on the HRV data, presenting a more complete picture of the environment in which rates of elevated and diminished stress response occurs.

### Mixed-Model Trajectory Analysis

Mixed-model trajectory analysis will be used to quantify the participant’s time series data. Maximum likelihood estimation and fit statistics tests will show which elements of the model improve fit to the observed data. This will help to control for biased estimates in a small sample size.

### Feasibility Analysis

Feasibility of the study protocol will be analyzed by determining the number and type of missed or incompletely collected data points. If data points fall outside of the normal range as found for previously reported field collection of biometric, salivary, and observational data collection, necessary protocol amendments will be made. Participants will also be interviewed postseason, at the completion of data collection, in order to identify challenges they may have had with the study protocol or use of the biometric device or salivary collection itself.

### Sample Size and Power

The implications of this study are limited by a small sample size of 10. However, given the study objective of determining the feasibility of using a personal wearable biometric device technology to study the physical and psychological occupational stress endured by commercial gillnet salmon fisherman in Bristol Bay, Alaska, the authors feel that the sample size does not present a limiting factor in achieving the study goal.

## Results

Data collection was initiated in July 2017 and will extend through August 2019. Data analysis will take place in fall 2019, and results will be disseminated via peer-reviewed publications in winter 2019. We anticipate that, if successful, this protocol will produce findings that could contribute substantially to the existing knowledge base regarding occupational health within the commercial fishing industry. Such findings would provide a foundation from which to revisit industry safety standards and an evidence base for future occupational health interventions.

## Discussion

We anticipate that the use of a biometric device will be crucial in establishing measures of stress and physical activity within a population and environment uniquely challenged by physical isolation, strong weather patterns, and the potential for significant financial gain by fishermen. This information can assist industry leaders in addressing sustained high rates of mortality within the industry and inform interventions to improve the health and well-being of fishermen. The primary limitation of the protocol is a small sample size, necessitated by the in-depth methods and physical isolation of study participants. We feel, however, that the high potential for knowledge to be gained justifies this limitation.

## Abbreviations

ECG: electrocardiograph
HPA: hypothalamic-pituitary-adrenal
HRV: heart rate variability
PPG: photoplethysmograph
UNC: University of North Carolina
